# Multicentre study on peri- and postoperative central venous oxygen saturation in high-risk surgical patients

**DOI:** 10.1186/cc5094

**Published:** 2006-11-13

**Authors:** Collaborative Study Group on Perioperative ScvO2 Monitoring

## Abstract

**Introduction:**

Low central venous oxygen saturation (ScvO_2_) has been associated with increased risk of postoperative complications in high-risk surgery. Whether this association is centre-specific or more generalisable is not known. The aim of this study was to assess the association between peri- and postoperative ScvO_2 _and outcome in high-risk surgical patients in a multicentre setting.

**Methods:**

Three large European university hospitals (two in Finland, one in Switzerland) participated. In 60 patients with intra-abdominal surgery lasting more than 90 minutes, the presence of at least two of Shoemaker's criteria, and ASA (American Society of Anesthesiologists) class greater than 2, ScvO_2 _was determined preoperatively and at two hour intervals during the operation until 12 hours postoperatively. Hospital length of stay (LOS) mortality, and predefined postoperative complications were recorded.

**Results:**

The age of the patients was 72 ± 10 years (mean ± standard deviation), and simplified acute physiology score (SAPS II) was 32 ± 12. Hospital LOS was 10.5 (8 to 14) days, and 28-day hospital mortality was 10.0%. Preoperative ScvO_2 _decreased from 77% ± 10% to 70% ± 11% (*p *< 0.001) immediately after surgery and remained unchanged 12 hours later. A total of 67 postoperative complications were recorded in 32 patients. After multivariate analysis, mean ScvO_2 _value (odds ratio [OR] 1.23 [95% confidence interval (CI) 1.01 to 1.50], *p *= 0.037), hospital LOS (OR 0.75 [95% CI 0.59 to 0.94], *p *= 0.012), and SAPS II (OR 0.90 [95% CI 0.82 to 0.99], *p *= 0.029) were independently associated with postoperative complications. The optimal value of mean ScvO_2 _to discriminate between patients who did or did not develop complications was 73% (sensitivity 72%, specificity 61%).

**Conclusion:**

Low ScvO_2 _perioperatively is related to increased risk of postoperative complications in high-risk surgery. This warrants trials with goal-directed therapy using ScvO_2 _as a target in high-risk surgery patients.

## Introduction

Several randomised controlled clinical studies have shown improved morbidity and mortality in high-risk surgical patients with perioperative optimisation of haemodynamics using strict treatment protocols in the single-centre setting [1-3]. The haemodynamic endpoints in goal-directed studies have been based on values derived from the pulmonary artery catheter [1-4], oesophageal Doppler [5-10], or (very recently) lithium indicator dilution and pulse power analysis [11]. Central venous oxygen saturation (ScvO_2_) and mixed venous oxygen saturation (SvO_2_) have been proposed to be indicators of the oxygen supply/demand relationship. However, the relationship between SvO_2 _and ScvO_2 _remains controversial [12]. Venous oxygen saturations differ among organ systems because different organs extract different amounts of oxygen. It is therefore conceivable that venous oxygen saturation depends on the site of measurement [13]. Redistribution of blood flow and alterations in regional oxygen demand (for example, in shock, severe head injury, general anaesthesia, as well as microcirculatory disorders) may affect the difference between ScvO_2 _and SvO_2_. Although ScvO_2 _principally reflects the relationship of oxygen supply and demand, mainly from the brain and the upper part of the body [13], it correlates reasonably well with concomitantly measured SvO_2 _[12,13], which is more dependent on changes in oxygen extraction in the gastrointestinal tract.

In patients with severe sepsis and septic shock, early ScvO_2_-driven haemodynamic treatment was found to reduce mortality [14]. More recently, low postoperative ScvO_2 _values were associated with an increased risk of complications in high-risk surgical patients [11]. Despite increasing evidence of beneficial effects on outcome, goal-directed therapies are not widely used in clinical practice. The reasons are a lack of demonstrated effect in large multicentre studies, the need for postoperative intensive care resources, the necessity of instituting complex protocols, as well as the need for monitoring techniques that are not routinely used in these specific patient groups. Using the ScvO_2 _as a potential target variable for haemodynamic optimisation is attractive because central venous catheterisation is routinely used in high-risk patients undergoing major surgery, ScvO_2 _can be screened, pre-emptive treatment is possible, and no major changes are necessary for the infrastructure in the operation area.

The present investigation was a pilot study designed to assess the incidence of low perioperative ScvO_2 _in high-risk surgical patients and the association of low ScvO_2 _with outcome in a multicentre setting. The aim was to evaluate whether the association of ScvO_2 _and postoperative complications in a strictly protocolised, interventional single-centre study on goal-directed haemodynamic management in high-risk surgical patients [11] could be confirmed in a purely observational, multicentre setting. Specifically, this pilot study was designed to clarify (a) the recruitment rate of patients scheduled for major surgery in a multicentre setting, (b) the range of perioperative ScvO_2 _in such patients, (c) the number of postoperative complications, and (d) the potential association between ScvO_2 _and complications. With these data, it should be possible to define whether a trial with goal-directed therapy using ScvO_2 _as a target is reasonable to conduct.

## Materials and methods

Two university hospitals in Finland and one in Switzerland participated in the study. The study was approved by the appropriate ethics committee for each institution, and written informed consent was obtained from each patient. Patients were screened for inclusion and exclusion criteria between September 20 and December 20, 2004.

### Inclusion criteria

For a patient to be included in the study, both of the following criteria had to be fulfilled: (a) increased surgical risk based on intra-abdominal or retroperitoneal surgery with an expected duration of at least 90 minutes or on abdominal aortic surgery and (b) two or more of Shoemaker's criteria of high risk [2]. These criteria include patient history (more than 70 years old with limited major physiological function, previous severe cardiopulmonary or vascular illness, and severe nutritional disorders), current clinical condition (severe multiple trauma, massive acute blood loss, shock, septicaemia or septic shock, respiratory failure, acute abdominal catastrophe, and acute intestinal or renal failure), the surgical procedure (extensive surgery for cancer or prolonged surgery more than eight hours), ASA (American Society of Anesthesiologists) class of greater than two, and a perioperative need for a central venous catheter.

### Exclusion criteria

Exclusion criteria for the study were a contraindication for a central venous catheter, unstable angina pectoris, primary hepatic or hepato-biliary surgery, the refusal of blood products, and the inability to give informed consent or refusal to consent.

### Study protocol

Anaesthesia, operation, and postoperative treatment were performed according to the local standards. All patients were postoperatively admitted either to an intensive care unit (ICU) or another high-dependency care (HDC) area (intermediate care unit or postanaesthesia care unit). Blood samples for the measurement of ScvO_2 _and haemoglobin were taken after induction of anaesthesia and thereafter at two hour intervals up to 12 hours postoperatively. Blood gas analyses were performed by intermittent blood sampling and co-oximetry (ABL 725; Radiometer, Copenhagen, Denmark [centres 1 and 2]; GEM Premier 3000; Instrumentation Laboratory, Barcelona, Spain [centre 3]).

### Complications

Complications and deaths occurring within 28 days of enrolment were included in the data analysis. Complications were prospectively defined and were diagnosed by clinical staff. Length of stay in the study hospital was censored at 28 days, and the patient's location at 28 days was recorded.

### Statistics

Data are presented as mean ± standard deviation when normally distributed, as medians (interquartile range) when not normally distributed, or (for categorical variables) as a percentage of the group from which they were derived. Normality was tested with the Kolmogorov-Smirnov test. Categorical data were tested with Fisher's exact test. Continuous data were tested with the *t *test when normally distributed and with the Mann-Whitney *U *test when not normally distributed. Trends in physiological parameters over time were compared with repeated-measures analysis of variance.

Univariate analysis was performed to test associations with complications and death. For data recorded hourly during the study period, the baseline values, the lowest values, and the mean over the 12-hour study period were tested. A multiple logistic regression model was used to identify independent risk factors for postoperative complications. A stepwise approach was used to enter new terms into the logistic regression model, where *p *< 0.05 was set as the limit for inclusion of new terms. Results of logistic regression are reported as adjusted odds ratios (ORs) with 95% confidence intervals (CIs). Receiver operator characteristic (ROC) curves were constructed to identify optimal cutoff values for association with outcome. The optimum cutoff was defined as the value associated with the highest sum of sensitivity and specificity. Analysis was performed with SPSS version 12.01 (SPSS Inc., Chicago, IL, USA) and Sigma Plot version 10.01 (Systat Software, Inc., Richmond, CA, USA) software, and significance was set at *p *< 0.05.

## Results

Of 218 screened patients, 60 patients fulfilled requirements for both inclusion and exclusion criteria and gave written informed consent (21 females, 39 males). Their mean age was 72 ± 10 years, and simplified acute physiology score (SAPS II) was 32 ± 12. In two centres, all patients were elective surgical cases, whereas in the third centre, 12 out of 21 patients were emergencies. Demographics and outcome data as well as indications for laparatomy stratified for the three centres are indicated in Table 1. Mean SAPS II scores in the three centres were 30, 26, and 40, respectively, and the associated mortality rates were 0%, 6%, and 24%, respectively.

As compared with preoperative values, ScvO_2 _was lower immediately after surgery. Haemoglobin decreased (preoperative 110 ± 19 g/l versus 102 ± 17 g/l immediately after surgery, *p *= 0.003).

Overall length of stay in the ICU/HDC was 1.0 (0 to 1) days, and hospital LOS was 10.5 (8 to 14) days (observation period 28 days). Six patients died (28-day mortality 10%); three of them were emergency cases (28-day mortality in emergency cases 25%).

**Figure 1:**
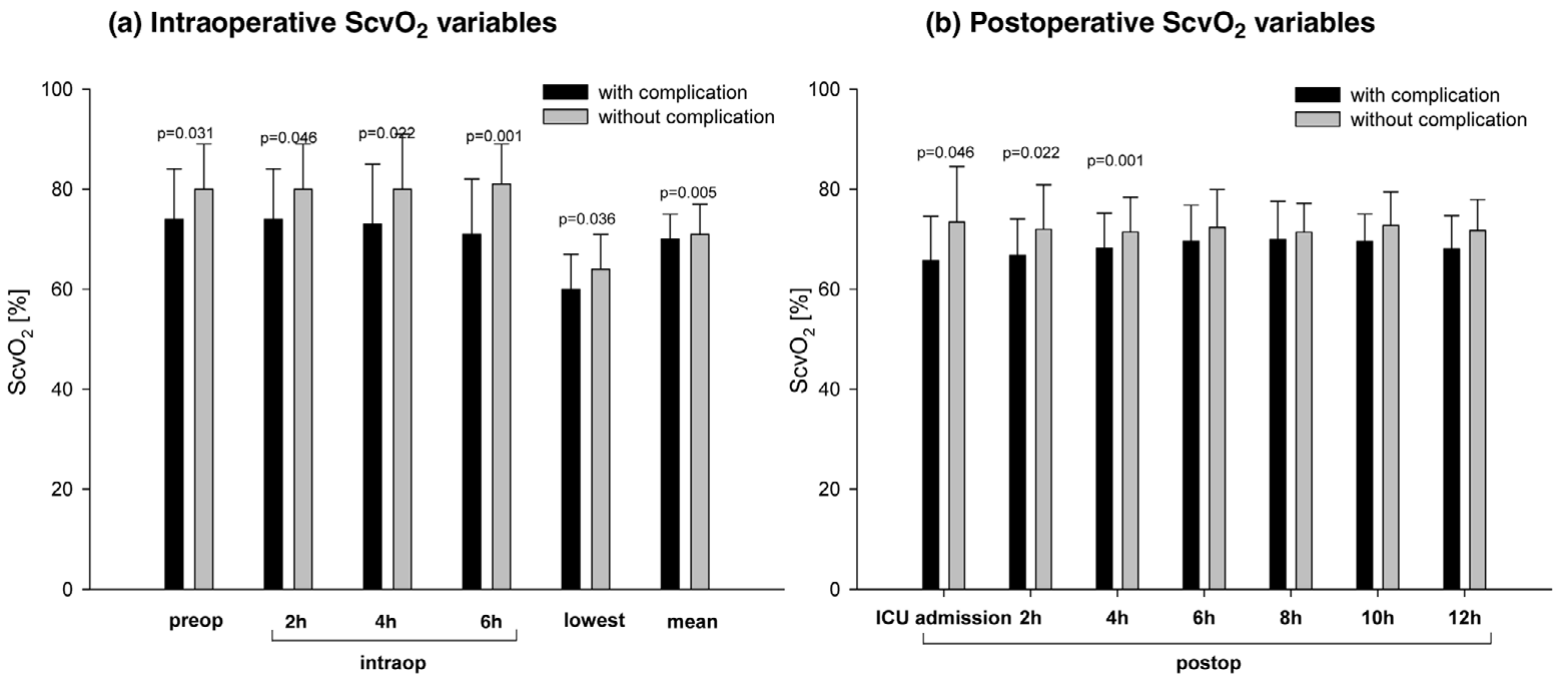
Intraoperative **(a) **and postoperative **(b) **ScvO_2 _variables in patients who did and did not develop postoperative complications. *P *values correspond to univariate analysis of variance. ICU, intensive care unit; IMC, intermediate care unit; intraop, intraoperative; preop, preoperative; postop, postoperative; ScvO_2_, central venous oxygen saturation.

Sixty-seven postoperative complications were recorded in 32 patients (20 cardiorespiratory, 23 surgical, 19 infectious, and 5 other; between 1.0 and 1.3 per patient per centre). Univariate analysis identified nine variables associated with postoperative complications (Table 2). Six of them were ScvO_2 _variables (Figure 1a,b). Additionally, haemoglobin (111 ± 18 versus 105 ± 23 g/l, *p *= 0.018), SAPS II (27 ± 11 versus 45 ± 26, *p *= 0.003), and hospital LOS (10 [8 to 12] versus 14 [10 to 17] days, *p *= 0.001) were associated with postoperative complications.

**Figure 2:**
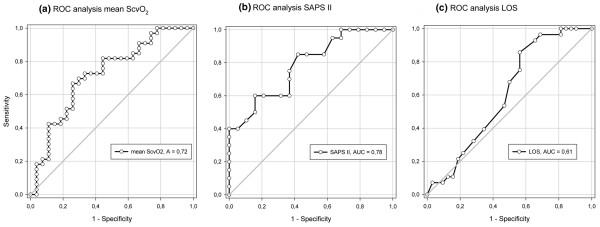
Receiver operator characteristic (ROC) analysis for **(a) **mean ScvO_2_, **(b) **SAPS II score, and **(c) **hospital length of stay (LOS). Outcome parameter for ROC analysis is occurrence of postoperative complications. Area under the curve (AUC) was 0.74 for mean ScvO_2 _(*p *= 0.004), 0.78 for SAPS II score (*p *= 0.002), and 0.61 for LOS (*p *= 0.15). SAPS II, simplified acute physiology score; ScvO_2_, central venous oxygen saturation.

**Figure 3:**
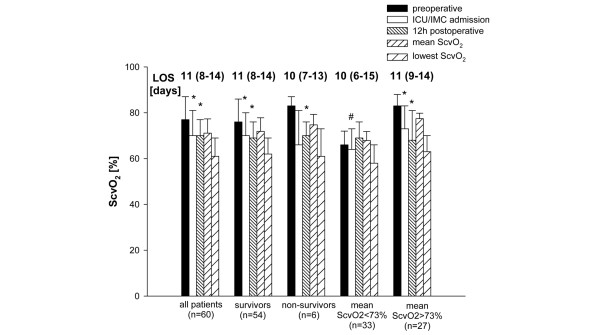
Central venous oxygen saturation (ScvO_2_) (percentage) in survivors and non-survivors and in patients with high and low mean ScvO_2 _values. The numbers above the error bars indicate the corresponding LOS data for the different sub-groups. *Wilcoxon signed rank test versus preoperative (*p *< 0.05). #Mann-Whitney test versus ScvO_2 _>73% (*p *= 0.001). ICU/IMC, intensive care unit/intermediate care unit; LOS, length of stay in hospital.

After multivariate analysis, mean ScvO_2 _value (OR 1.23 [95% CI 1.01 to 1.50], *p *= 0.037), hospital LOS (OR 0.75 [95% CI 0.59 to 0.94], *p *= 0.012), and SAPS II (OR 0.90 [95% CI 0.82 to 0.99], *p *= 0.029) were independently associated with postoperative complications. ROC curves for these variables are displayed in Figure 2. The areas under the ScvO_2 _and SAPS II, but not LOS, ROC curves were significantly different from 0.5 (*p *= 0.004 and 0.002, respectively). The optimal value of mean ScvO_2 _for discriminating between patients who did or did not develop complications was 73% (sensitivity 72%, specificity 61%). The relation between ScvO_2 _and hospital LOS in survivors and non-survivors is displayed in Figure 3.

## Discussion

The main finding of this study was that in the multicentre setting, low ScvO_2 _during the peri- and postoperative period was associated with an increased risk of postoperative complications in high-risk patients undergoing major surgery. Our results support the feasibility of testing ScvO_2 _as a target variable to improve outcome in high-risk surgery. The criteria to define patients at high risk were pragmatic and clinically oriented and resulted in a sufficient recruitment rate. Furthermore, despite the relative heterogeneity of the patient population, ScvO_2 _had a reasonable predictive value for postoperative complications.

Pearse *et al*. [11] found that low minimum ScvO_2 _values during the first eight postoperative hours were associated with increased risk of postoperative complications. Their findings come from a strictly protocolised, interventional single-centre study on goal-directed haemodynamic management in high-risk surgical patients. Our results further confirm the association between ScvO_2 _and postoperative complications in a purely observational, multicentre setting. Furthermore, intraoperatively, we were able to demonstrate a significant difference in ScvO_2 _between patients who did and did not develop complications. Taken together, the present study and that of Pearse *et al*. suggest that the overall peri- and postoperative course of ScvO_2 _should be taken into account if ScvO_2_-targeted interventions are considered for testing in large-scale clinical trials.

Despite the relative similarity of the participating centres and the presence of comparable infrastructures for postoperative care, the hospital LOS varied widely between the centres. The reason for this is certainly multifactorial and likely to include, among other things, care processes within the individual centres, discharge policies, and variations in local health care organisation. These variations were likely to dilute any association between ScvO_2 _and length of stay in the relatively small sample size. Hence, we used clinically relevant predefined complications as the main outcome measure. The hospital mortality in the present study was comparable with the recent study of Pearse *et al*. [11] and clearly lower than what would be expected from several previous studies on high-risk surgery. Due to the small sample size and different proportions of emergency patients, relevant between-centre comparisons cannot be made.

The prognostic significance of ScvO_2 _less than 65% has been demonstrated in myocardial infarction [15], trauma [16], severe sepsis [17], and cardiac failure [18]. However, the only interventional trial of ScvO_2 _conducted so far used a target of 70% [14]. In the study of Pearse *et al*. [11], a level of 65% seemed to discriminate best between patients with and without complications. This may be related to the lower tissue oxygen delivery in surgical patients as compared with patients with sepsis. Despite complex physiology, the association between ScvO_2 _and outcome after major surgery seems to be similar to the association between cardiac index and outcome or between oxygen delivery and outcome [19-22].

The best cutoff for ScvO_2 _in predicting complications in our study was 73%. This corresponds well with the mean ScvO_2 _of 75% found by Pearse *et al*. [11] in patients who did not develop complications. The observed cutoff value of ScvO_2 _should be interpreted with some caution due to the sample size. Nevertheless, the somewhat higher best cutoff of ScvO_2 _for predicting complications in our study could be related to the fact that our study was observational, whereas Pearse *et al*. used protocolised treatment. When using protocols, the fluctuation in ScvO_2 _is likely to be reduced. This may also explain why in our study the mean ScvO_2_, rather than the minimum ScvO_2_, had predictive value.

In our study, the perioperative mean of ScvO_2 _was 74% in patients who did not develop postoperative complications. This is comparable with previous measurements in healthy subjects [23] and in patients after surgery [11,22] but is higher than in patients with favourable outcome after severe sepsis, trauma, cardiac failure, or myocardial infarction [15-18]. Accordingly, targets for ScvO_2 _in future prospective trials should probably be adapted to the specific study groups.

We believe that our results encourage trials with goal-directed therapy using ScvO_2 _as a target in high-risk surgery patients. Based on our data and in agreement with results from others [11], target values should be in the range of 70% to 75%, and values less than 65% should be strictly avoided. In patients with cardiac failure or trauma, lower targets (at approximately 65%) may be appropriate [15,16].

Obviously, in a multicentre approach, the inclusion of 60 patients with an observed 28-day mortality of 10% is enough to be able to demonstrate a benefit in terms of complication rate but not in terms of length of stay and mortality. To demonstrate a relative reduction in 28-day mortality of 34% (as in the study of Rivers *et al*. [14], with a beta error of 80% and an alpha error of 5%, the sample size in a patient group similar to that in this study would be 85 for both groups. Although the risk factors for postoperative complications agree well with previous studies, the small sample size for the multivariate analysis should be considered in interpreting our results.

Because oxygen demands are normally well controlled during general anaesthesia, efforts to increase ScvO_2 _should target oxygen delivery (arterial oxygen saturation, haemoglobin, and cardiac output). In fact, in this trial, preoperative haemoglobin concentrations were significantly lower in patients with complications as compared with patients without. To avoid sudden drops in ScvO_2 _as a consequence of the combination of hypovolaemia and anaemia during surgical bleeding, it may be prudent to correct low (<10 g/dl) preoperative haemoglobin concentrations.

A drop in ScvO_2 _was noted between the end of surgery and the first readings in the ICU. This finding is consistent with previous findings on ScvO_2 _[11] and SvO_2 _[19,21] in surgical patients. Both decreased systemic oxygen delivery and increased oxygen consumption may have contributed. Pearse *et al*. [11] reported unchanged cardiac output in the postoperative period. If this was the same in our patients, oxygen delivery still could have decreased due to the significantly lower postoperative haemoglobin concentrations. Postoperative oxygen consumption is determined by various factors, including pain, emergence from anaesthesia, body temperature, and shivering. To avoid low postoperative ScvO_2_, all of these factors may have to be controlled.

## Conclusion

In our study, low ScvO_2 _was frequently observed in patients during and after major surgery and was related to postoperative complications. In prospective trials using ScvO_2 _as a goal, the specific patient group has to be taken into account when target levels are defined.

## Key messages

• Low ScvO_2 _perioperatively is related to increased risk of postoperative complications in high-risk surgery.

• Trials with goal-directed therapy using ScvO_2 _as a target in high-risk surgery patients are warranted.

## Abbreviations

HDC = high-dependency care; ICU = intensive care unit; LOS = length of stay; OR = odds ratio; ROC = receiver operator characteristic; SAPS II = simplified acute physiology score; ScvO_2 _= central venous oxygen saturation; SvO_2 _= mixed venous oxygen saturation.

## Competing interests

The authors declare that they have no competing interests. No author received individual funding in connection with this study.

## Authors' contributions

HB performed programming of databases for all centres, data acquisition, data analysis, calculation of statistics and manuscript revision. VE, DI, SL, IP, CR, and AV provided data acquisition and manuscript revision. MH carried out data acquisition and interpretation and manuscript revision. SMJ participated in the study design and coordination, performed the measurements, and wrote a first draft of the manuscript. HL and SN recruited patients, performed data acquisition, data analysis and interpretation, and manuscript revision. KM and PM carried out patient recruitment, data acquisition, and manuscript revision. MN and ER performed data interpretation and manuscript revision. JT provided study conception and design, data interpretation, and manuscript revision. All authors were given the opportunity to read and approve the final manuscript.

## Appendix

The Collaborative Study Group on Perioperative ScvO_2 _Monitoring is composed of authors from three centres:

Bern:

Hendrik Bracht^1^, Verena Eigenmann^2^, Matthias Haenggi^1^, Daniel Inderbitzin^3^, Stephan M Jakob^1 ^(co-principal investigator), Stefanie Loher^1^, Christine Raeber^1^, Jukka Takala^1 ^(coordinating investigator), Andreas Vogt^7^

Kuopio:

Kimmo Mäkinen^5^, Pekka Miettinen^5^, Minna Niskanen^6^, Ilkka Parviainen^6 ^(co-principal investigator)

Tampere:

Heli Leppikangas^4^, Silvia Nunes^4^, Esko Ruokonen^4 ^(co-principal investigator)

^1^Department of Intensive Care Medicine, University Hospital Bern, CH-3010 Bern, Switzerland

^2^Department of Cardiovascular Surgery, University Hospital Bern, CH-3010 Bern, Switzerland

^3^Department of Visceral and Transplantation Surgery, University Hospital Bern, CH-3010 Bern, Switzerland

^4^Department of Intensive Care, Tampere University Hospital, P.O. Box 2000, 33521 Tampere, Finland

^5^Department of Surgery, Kuopio University Hospital, P.O. Box 1777, 70211 Kuopio, Finland

^6^Department of Anesthesia and Intensive Care Medicine, Kuopio University Hospital, P.O. Box 1777, 70211 Kuopio, Finland

^7^Department of Anesthesiology, University Hospital Bern, CH-3010 Bern, Switzerland

## Figures and Tables

**Table 1:** Demographics and outcome data stratified for the three centres

	All centres (*n *= 60)	Centre 1 (*n *= 17)	Centre 2 (*n *= 22)	Centre 3 (*n *= 21)
Age in years	72 ± 10	68 ± 11	72 ± 9	74 ± 10
Male/female	40/20	13/4	18/4	16/5
SAPS II^a^	32 ± 12	26 ± 10	30 ± 4	40 ± 14
Emergency surgery (percentage)	20	0	0	57
Aortic/iliacal aneurysm/dissection (percentage)	47	82	50	14
Carcinoma upper abdomen (percentage)	12	12	5	5
Carcinoma lower abdomen (percentage)	24	6	41	38
Infection (percentage)	7	0	4	19
Other (percentage)	10	0	0	24
Duration of operation (minutes)	218 ± 111	323 ± 92	178 ± 81	177 ± 99
Hospital LOS (days)	11 (8 to 14)	13 (11 to 15)	8 (4 to 10)	11 (9 to 14)
Patients at home on day 28 (percentage)	65	23	90	38
Patients in hospital/nursing facility on day 28 (percentage)	22	65	5	38
Unknown patient location on day 28 (percentage)	3	6	5	0
Mortality (percentage)	10	6	0	24

^a^One-way analysis of variance (*p *< 0.01). LOS, length of stay; SAPS II, simplified acute physiology score.

**Table 2:** Variables associated with postoperative complications

	Patients with complications (*n *= 32)	Patients without complications (*n *= 28)	*P *value^a^
ScvO_2 _(percentage)			
Preoperative	74 ± 10	80 ± 9	0.031
Intraoperative			
After 1 hour of surgery	74 ± 10	80 ± 9	0.046
After 2 hours of surgery	73 ± 12	80 ± 11	0.022
After 3 hours of surgery	71 ± 11	81 ± 8	0.001
Lowest	60 ± 7	64 ± 7	0.036
Mean	70 ± 5	74 ± 6	0.005
Haemoglobin at ICU admission (g/l)	95 ± 17	105 ± 13	0.018
SAPS II	41 ± 14	27 ± 11	0.003
Hospital LOS (days)	13 ± 7	10 ± 4	0.001

^a^Univariate analysis of variance. ICU, intensive care unit; LOS, length of stay; SAPS II, simplified acute physiology score; ScvO_2_, central venous oxygen saturation.
